# Absence of CD11a Expression Identifies Embryonic Hematopoietic Stem Cell Precursors *via* Competitive Neonatal Transplantation Assay

**DOI:** 10.3389/fcell.2021.734176

**Published:** 2021-08-25

**Authors:** Alborz Karimzadeh, Erika S. Varady, Vanessa M. Scarfone, Connie Chao, Karin Grathwohl, Pauline U. Nguyen, Yasamine Ghorbanian, Irving L. Weissman, Thomas Serwold, Matthew A. Inlay

**Affiliations:** ^1^Sue and Bill Gross Stem Cell Research Center, University of California, Irvine, Irvine, CA, United States; ^2^Department of Molecular Biology and Biochemistry, University of California, Irvine, Irvine, CA, United States; ^3^Institute of Stem Cell Biology and Regenerative Medicine and Ludwig Center, Stanford University, Stanford, CA, United States; ^4^Joslin Diabetes Center, Harvard Medical School, Boston, MA, United States

**Keywords:** embryo, flow cytometry, hematopoietic stem cell (HSC), hematopoietic stem cell transplantation, neonatal transplantation, embryonic hematopoiesis

## Abstract

Hematopoietic stem cells (HSCs) are defined by their self-renewal, multipotency, and bone marrow (BM) engraftment abilities. How HSCs emerge during embryonic development remains unclear, but are thought to arise from hemogenic endothelium through an intermediate precursor called “pre-HSCs.” Pre-HSCs have self-renewal and multipotent activity, but lack BM engraftability. They can be identified functionally by transplantation into neonatal recipients, or by *in vitro* co-culture with cytokines and stroma followed by transplantation into adult recipients. While pre-HSCs express markers such as Kit and CD144, a precise surface marker identity for pre-HSCs has remained elusive due to the fluctuating expression of common HSC markers during embryonic development. We have previously determined that the lack of CD11a expression distinguishes HSCs in adults as well as multipotent progenitors in the embryo. Here, we use a neonatal transplantation assay to identify pre-HSC populations in the mouse embryo. We establish CD11a as a critical marker for the identification and enrichment of pre-HSCs in day 10.5 and 11.5 mouse embryos. Our proposed pre-HSC population, termed “11a- eKLS” (CD11a- Ter119- CD43+ Kit+ Sca1+ CD144+), contains all *in vivo* long-term engrafting embryonic progenitors. This population also displays a cell-cycle status expected of embryonic HSC precursors. Furthermore, we identify the neonatal liver as the likely source of signals that can mature pre-HSCs into BM-engraftable HSCs.

## Introduction

Hematopoietic stem cells (HSCs) in adults are the multipotent and self-renewable source of the entire blood system, and hold the regenerative capacity to engraft a myeloablated recipient upon transplantation (Hagedorn et al., 2014). While the identity, self-renewal ability, engraftment potential, and differentiation properties of adult HSCs has been extensively studied over the past 30 years, much less is known about the developmental origins of HSCs in the embryo. During early embryonic development and prior to the appearance of fully-functional HSCs, distinct waves of blood-forming cells emerge, likely initiated from specialized endothelial cells called “hemogenic endothelium” (Zovein et al., 2008; Lancrin et al., 2009). These waves overlap, with each wave functionally more mature than the last. In mice, the initial wave of hematopoiesis gives rise primarily to primitive nucleated erythrocytes and arises in the yolk sac (YS) blood islands starting from embryonic day (e) 7.5 (Moore and Metcalf, 1970). After establishment of a heartbeat, definitive hematopoiesis begins at e8.5 in the YS and placenta (PL) with a transient wave of erythro-myeloid progenitors (Palis et al., 1999; Alvarez-Silva et al., 2003; Palis, 2016). At e9.5, the first self-renewable and multipotent progenitors, that immediately precede HSCs, emerge in the YS, aorta-gonad-mesonephros (AGM), and PL, and are often called “pre-HSCs” (Yoder et al., 1997a; Taoudi et al., 2008; Arora et al., 2014; Inlay et al., 2014; Rybtsov et al., 2016; Zhou et al., 2016). After e11.5, the pre-HSC wave transitions into an expanding BM-engraftable HSC pool in the fetal liver (FL) (Kumaravelu et al., 2002; Gekas et al., 2005). The FL remains the major site of hematopoiesis until perinatal seeding of the BM (Muller et al., 1994; Beaudin et al., 2016).

In adult mice, multi-parameter fluorescence-activated cell sorting (FACS) coupled with transplantation assays have enabled the isolation of a highly purified bone marrow (BM) HSC population for functional and molecular characterization (Wiesmann et al., 2000; Kiel et al., 2005; Balazs et al., 2006; Karlsson et al., 2013). BM HSCs can be sorted, then transplanted intravenously into lethally-irradiated wild-type recipient mice, where they will home to the BM, engraft, and reconstitute the hematopoietic system for the life of the recipient. Pioneering work on the embryonic origins of HSCs led to the present definition of pre-HSCs as cells that have the self-renewal and lineage potential of adult HSCs, but lack the ability to engraft into the BM when transplanted intravenously into adult lethally-irradiated recipients (Weissman et al., 1978; Akashi and Weissman, 2001). However, two alternative assays have been developed to functionally identify pre-HSC activity in the embryo: *ex vivo* maturation and neonatal transplantation. In the former, candidate populations or tissues are harvested from the embryo and cultured *in vitro* with the addition of exogenous factors to induce *ex vivo* maturation of these cells into HSCs, which is then confirmed by adult transplantation (Taoudi et al., 2008; Rybtsov et al., 2011). However, these *ex vivo* maturation assays rely on the presence of cultured stromal lines as well as potent exogenous factors such as SCF, TPO, IL-3, and Flt3L. Accordingly, these assays can potentially drive HSC formation from cell-types that are more primitive than pre-HSCs, such as hemogenic endothelium (Hadland et al., 2017). An alternative approach to reveal pre-HSC activity is *via* intravenous injection of embryonic cells directly into irradiated *neonatal* recipients (Yoder and Hiatt, 1997; Yoder et al., 1997b,a). While less sensitive than *ex vivo* cultures, neonatal transplantation presents minimal risk of introducing artifacts by bypassing the non-physiological concentrations of cytokines and growth factors used *ex vivo* (Yoder et al., 1997a; Boisset et al., 2010; Arora et al., 2014).

Adult HSCs can be precisely identified by a combination of different markers expressed (or unexpressed) on their surface. While many different combinations can work, a commonly used definition for murine HSCs is Lineage- Kit+ Sca1+ CD150+ and CD34-. However, many adult HSC markers are not similarly expressed in the early embryo and can change depending on the tissue and timepoint examined (Cumano and Godin, 2007). Alternative assays have identified potential pre-HSC markers including hematopoietic markers CD41 (Rybtsov et al., 2011), CD43 (Inlay et al., 2014), and CD45 (Taoudi et al., 2008; Boisset et al., 2010), progenitor markers Kit (Boisset et al., 2010) and Sca1 (Inlay et al., 2014), and endothelial markers CD31 (Inlay et al., 2014), VE-Cadherin (CD144) (Taoudi et al., 2008), and EPCR (CD201) (Zhou et al., 2016). This has resulted in the identification of populations such as Type I (CD144+ CD41+ CD45-) and Type II (CD144+ CD45+) pre-HSCs (Rybtsov et al., 2011), or rarer CD201^hi^ subsets within these populations (Zhou et al., 2016) or a CD27+ subset within Type II pre-HSCs (Li et al., 2017). However, a strictly-defined pre-HSC cell type has not been described to the same resolution as that in adult HSCs.

CD11a (integrin alpha L, or *Itgal*) forms the complex LFA-1 (leukocyte functional-associated antigen 1; α_L_β_2_) upon dimerization with the β2-integrin CD18. LFA-1 interacts with ICAMs and has roles in lymphocyte activation, differentiation, and transendothelial migration (Kinashi, 2005; Shamri et al., 2005; Zhang and Wang, 2012). CD11a is highly expressed on all circulating immune cells, including BM progenitor populations (Fathman et al., 2014). However, our previous work found CD11a to be uniquely unexpressed in a subset of adult HSCs (defined as Lin- Kit+ Sca1+ CD150+ CD34-), and only the CD11a- fraction of adult HSCs displayed long-term multilineage reconstitution upon transplantation (Fathman et al., 2014; Karimzadeh et al., 2018). In a related study, we examined the potential of CD11a as a marker of embryonic multipotent progenitors in e9.5-11.5 embryos. Using a single-cell *in vitro* multipotency assay, we determined that only a rare CD11a- population we termed “CD11a- KLS” cells (defined as Ter119- CD43+ Kit+ Sca1+ CD144+ CD11a-) contained all multipotent progenitor activity, regardless of what timepoint or tissue it was isolated from Inlay et al. (2014). Neonatal transplantation demonstrated these cells produce a variety of lineages *in vivo*, though their long-term engraftment and ability to give rise to HSCs was never assessed. Thus, in both studies, the absence of CD11a expression was an important marker to identify adult HSCs by transplantation, and a candidate pre-HSC population in the embryo by *in vitro* multipotency.

In the present study, we use an *in vivo* neonatal NSG transplantation system to prospectively identify pre-HSCs in e10.5 and e11.5 tissues. In line with our previous work, the absence of CD11a expression on pre-HSCs (defined as Ter119- CD43+ Kit+ Sca1+ CD144+ CD11a-) was critical for distinguishing them from downstream progenitors which were all CD11a+. Moreover, our data suggest the neonatal liver serves as an essential temporary niche for the maturation of embryonic progenitors which lack the expression of the BM homing receptor CXCR4 prior to seeding the BM. These findings establish CD11a as a key marker to identify and isolate a highly purified pre-HSC population, beyond what has been achieved, therefore paving the way for more detailed characterization of these immature progenitors.

## Materials and Methods

### Antibodies

A detailed list of all antibodies used in this study is shown in Supplementary Table 1.

### Mice

In our experiments, we used embryos from a *Rosa26*^*Tomato/CFP*^ male crossed to a *Rosa26*^*wt/wt*^ (C57Bl/6; Jackson Laboratory; stock no. 00664) female. *Rosa26*^*Tomato/CFP*^ males were generated from a cross between *Rosa26*^*Tomato/Tomato*^ (mT/mG; Jackson Laboratory; stock no. 007576) and *Rosa26*^*CFP/CFP*^ (TM5; generous donation by Dr. Irving Weissman). NSG (NOD-*scid* IL-2Rγ^null^; Jackson Laboratory; Stock no. 005557) mice were used as neonatal recipients. All strains were maintained at the Gross Hall and Med Sci A vivarium facilities at UCI and fed with standard chow and water. All animal procedures were approved by the International Animal Care and Use Committee (IACUC) and University Laboratory Animal Resources (ULAR) of University of California, Irvine.

### Embryo Harvest and Tissue Processing

Mating cages were established and vaginal plugs were checked every morning to determine the time of pregnancy. The morning of plug detection was assigned as day 0.5. Pregnant mice were dissected and embryos harvested in PBS + 2% fetal bovine serum (FACS buffer) and kept on ice during tissue dissection. Somite pairs were counted and averaged for each experiment to determine dpc. Dpc designation is as follows: 15–29 somite pairs: e9.5; 30–39 somite pairs: e10.5; 40–50 somite pairs: e11.5. For tissue analyses and non-sorted transplants, CH, YS, and PL were harvested from e9.5 embryos. For e10.5 and e11.5 embryos, AGM and FL were harvested separately instead of together (e.g., CH). For sorted transplants, CH, YS, and PL were harvested from e10.5 donors and AGM, YS, PL, and FL from e11.5 donors. For non-sorted transplants, YS was harvested with the vitelline vessels, and PL was harvested with umbilical vessels. For sorted transplants, YS was separated without the vitelline vessels, and PL was harvested with umbilical vessels. Separated tissues were digested with 1 mg/mL Collagenase Type IV (ThermoFisher Scientific; cat. no. 17104019) for 30–45 min at 37°C. Tissues were pipetted up and down at 15-min intervals to aid with the digestion. Single cell suspension was filtered using a 40 μ mesh. We recommend using 40 μ (instead of 70 μ) mesh for donor cells to minimize clogging of blood vessels upon injection into neonatal recipients. Cells were washed twice and resuspended in FACS buffer for staining/transplantation.

### Cell Sorting

Single cell suspensions of cells were typically stained for 20–30 min on ice. We recommend using ACK lysis buffer *after* completion of cell staining as pre-staining use can affect the VE-Cadherin signal. For sorting, a BD FACS-Aria II (Becton Dickinson) with FACSDiva software was used. For sorted transplants, the “purity” mode was used for cell sorting. Since opposing populations from differentially labeled embryo cells were pooled together, only embryo batches with close to 50–50% color distribution were used for the competitive sorted transplant. Therefore, physiological ratios of opposing populations were reflected in the final tube to be transplanted. For short-term homing sorts, the “yield” mode was used for cell sorting to maximize cell recovery.

### *In vivo* Transplantation and Analysis

For non-sorted transplants, the embryo equivalent used for each timepoint is as follows: ≥4 ee for e9.5, ≤3 ee for e10.5, and ≤1 ee for e11.5. For all transplants, single cell suspensions were resuspended in 50–70 μL FACS buffer for injection with defined numbers of adult helper BM added. For neonatal transplants, cells were injected into the facial vein of sublethally irradiated (180–200 Rads; XRAD 320, Precision X-ray) P1-P4 NSG recipients. Nursing NSG mothers were fed an antibiotic chow of Trimethoprim Sulfa (Uniprim, Envigo) for 4 weeks post transplant to prevent bacterial infections. For secondary transplantation into adult recipients, recipient C57BL/6 mice were conditioned with 800 Rads, anesthetized by isoflurane, and retro-orbitally injected with 1–2 million whole BM harvested from primary recipients. For peripheral blood analysis, blood was obtained from the tail vein of transplanted mice at various timepoints, and red blood cells were depleted using ACK lysis buffer. For BM analysis, BM was harvested from tibias and femurs by flushing with ice-cold FACS buffer followed by ACK lysis and filtration. Cells were stained with lineage antibodies and analyzed on the BD FACS-Aria II. FlowJo software (Tree Star) was used for data analysis.

### Cell Cycle Analysis

FoxP3/Transcription Factor Staining Buffer Kit (Tonbo Biosciences; cat. no. TNB-0607) is a paraformaldehyde/saponin based fixation/permeabilization buffer set for intracellular staining, and was adapted here for cell cycle analysis. Briefly, cells stained with extracellular antibodies were fixed with 1X Tonbo Foxp3/Transcription Factor Fix/Perm buffer for 45 min at 4°C, permeabilized/stained with PE anti-ki-67 antibody (Biolegend; cat. no. 652403) diluted in 1X Tonbo Flow Cytometry Perm Buffer for 45 min in the dark at room temperature. Cells were then washed and stained with 1 μM DAPI (Biolegend; cat. no. 422801) for 10 min prior to flow cytometric analysis. Ki-67 is a nuclear protein associated with cellular proliferation, and is expressed on cells that have entered the cell cycle, but not on quiescent G0 cells (Kim and Sederstrom, 2015). DAPI is a nuclear dye that can distinguish cells that have undergone DNA replication. We recommend avoiding separation of e10.5 tissues into fewer than 4 ee as the fix/perm process results in loss of cells.

### Short-Term Homing Analysis

Neonatal recipients were sacrificed 15 h post-transplant for tissue dissection. Care was taken to harvest tissues in their entirety. All tissues except bones were harvested by crushing in between slides followed by separation using a 28-gauge needle. Limb bones were crushed using a pestle and mortar. Crushed bone particles were passed through a 28-gauge needle for further separation. All tissues were filtered through a 40 μ mesh and ACK lysed prior to staining.

### Statistical Analysis

Statistical analysis was performed with GraphPad Prism 5 software (La Jolla, CA, United States).

## Results

### Establishing the NSG Neonatal Transplant System for *in vivo* Detection of Pre-HSCs

**FIGURE 1:**
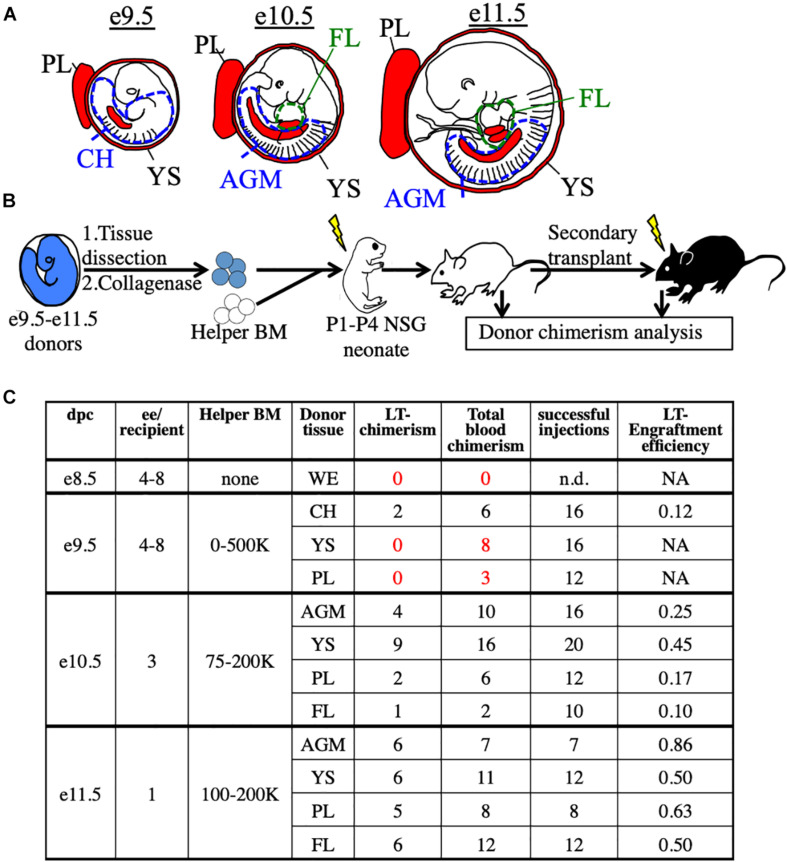
Utilization of the NSG neonatal transplant system to reveal pre-HSCs in e9.5–11.5 embryos. **(A)** Schematic representation of hematopoietic tissues (red) harvested from e9.5, e10.5, and e11.5 embryos. YS, yolk sac; PL, placenta; CH, caudal half; AGM, aorta-gonad-mesonephros; FL, fetal liver. Dissection sites of the CH/AGM and FL are shown in blue and green dashed lines, respectively. **(B)** Schematic representation of the neonatal transplant system. Harvested tissues from e9.5–11.5 donor CFP+ embryos are dissected, dissociated, and combined with CFP- adult helper BM. Donor cells are administered intravenously (i.v.) into irradiatated neonatal NSG recipients of 1–4 days of age (P1-P4). Recipients are bled for donor chimerism analysis at 4 week intervals and are then sacrificed for BM analysis and secondary transplants. **(C)** Compilation of whole-tissue transplants from e8.5–11.5 embryos. Number of successful tissue-specific long-term engraftment (“LT-chimerism”) is determined by the presence of ≥1% embryo donor granulocyte chimerism (at the last bleed) and HSPC (hematopoietic stem/progenitor cell) chimerism in the BM. “Total blood chimerism” refers to the number of recipients with ≥1% embryo chimerism in total CD45+ compartment of blood. “Successful injection” is defined as engraftment of either embryo or helper adult BM donors. “Engraftment efficiency” is determined by # of successful embryo engraftment/total successful injections. dpc, days post conception; ee, embryo equivalent; WE, whole embryo.

To functionally detect pre-HSC activity *in vivo*, we used immunodeficient NSG (NOD/SCID/IL2rγ**^–/–^**) neonatal mice as transplantation recipients (Ishikawa, 2013; Verbiest et al., 2016; Dai et al., 2017). We first tested this system on unsorted embryonic cells harvested from e9.5 to e11.5 tissues, the stages when pre-HSCs are thought to emerge, expand, and mature. From e9.5 embryos we harvested the YS, PL, and caudal half (CH), which contains the AGM. From e10.5 and e11.5 embryos we harvested the YS, PL, AGM, and FL (Figure 1A). Cells from whole tissues of e9.5–11.5 donors along with adult helper BM were transplanted into irradiated NSG neonates followed by tissue analysis and secondary transplants (Figure 1B and Supplementary Figure 1). While donor chimerism from e9.5 donors was detected at extremely low rates, we consistently detected BM HSC engraftment from all tissues of e10.5 and e11.5 donors (Figure 1C and Supplementary Figure 1). To confirm that none of the transplanted cells were *already* HSCs, we separately transplanted e10.5 and e11.5 donor cells into *adult* recipients and did not find any long-term adult BM engraftment (Supplementary Figure 2). It should be noted that while e11.5 tissues can contain adult-engraftable HSCs, this activity is rare, often less than one HSC per embryo (Arora et al., 2014), and we did not find any HSC-engraftment in our transplants. Therefore, any engraftment in the neonatal recipients must have come from cells which lack adult engraftability, and are thereby pre-HSCs.

### Establishment of an Embryonic Competitive Transplant System

**FIGURE 2:**
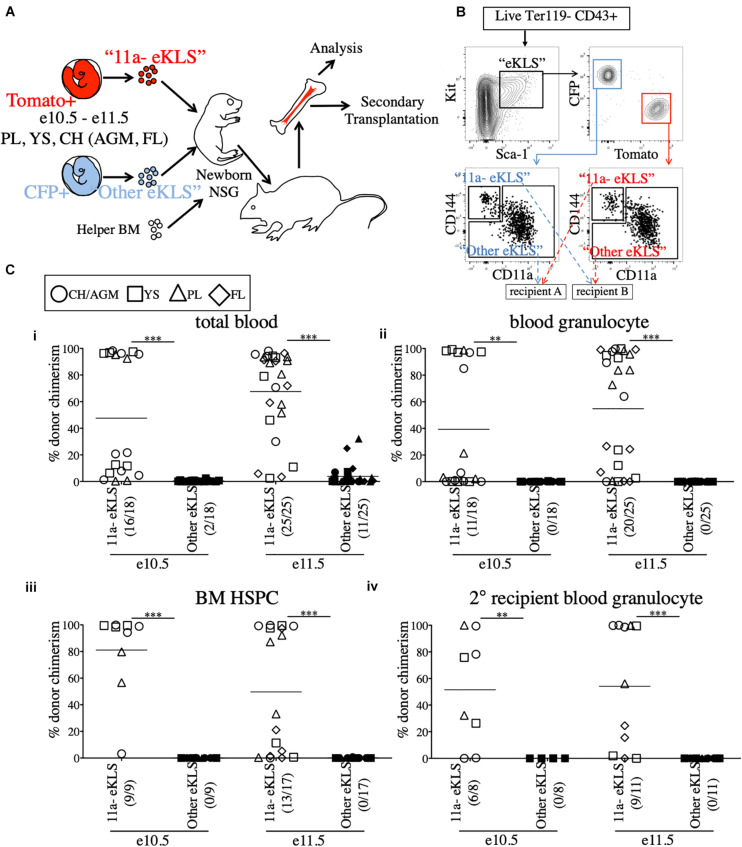
All functional pre-HSCs are contained within the CD11a- fraction of e10.5 and e11.5 embryonic progenitors. **(A)** Schematic representation of the competitive transplant system. Sorted Tomato-expressing “11a-eKLS” and CFP-expressing “Other eKLS” cells (and vice versa) are combined and mixed with non-fluorescent CD45.2+ adult helper BM for transplantation into newborn NSG recipients. **(B)** Representative sorting strategy for the competitive transplantation of 11a- eKLS and Other eKLS populations. Embryonic progenitors are defined as live, Ter119- CD43+ Kit+ Sca1+ cells. Tomato+ and CFP+ progenitors are then gated, and CD11a- CD144+ (“11a- eKLS”) and everything else (“Other eKLS”) are sorted. Opposing populations of different colors are mixed post-sort and transplanted into the same recipient. Representative plots and gates for each tissue and timepoint can be found in Supplementary Figure 3. **(C)** Donor chimerism from 11a- eKLS and Other eKLS populations in primary and secondary recipients. Percent donor chimerism of total blood **(i)**, blood granulocyte **(ii)**, BM HSPC **(iii)** in primary recipients and blood granulocyte chimerism in secondary recipients **(iv)** from 11a- eKLS (white symbols) and Other eKLS (black symbols). Numbers in parenthesis indicate successful chimerism/total recipients engrafted with embryonic cells of any source. Blood analysis was done after at least 12 weeks post primary transplant and after at least 6 weeks post secondary transplant. 1% is set as a threshold to define successful chimerism. CH/AGM (circle), YS (squares), PL (triangles), and FL (diamond) are shown. For a tissue-specific analysis of both timepoints, refer to Supplementary Figure 4.

We next used the NSG neonatal transplant system to assay sorted populations for pre-HSC activity. To allow us to directly compare sorted embryonic populations in a competitive setting, we bred mice to generate embryos that expressed either CFP or Tomato fluorescent reporters in the same litters. To accomplish this, males bearing two reporters, Tomato and CFP (*Rosa26*^*Tomato/CFP*^), on different alleles of the *Rosa26* locus, were crossed to Wt females (*Rosa26*^*wt/wt*^). Each offspring will receive only one reporter allele and be either Tomato+ (*Rosa26*^*Tomato/wt*^) or CFP+ (*Rosa26*^*CFP/wt*^). Therefore, age-matched littermates can be distinguished by color, sorted based on marker expression, and co-transplanted in a head-to-head competitive setting (Figures 2A,B). Provided equal numbers of embryos of each color are used, we can directly compare two populations to determine which contains more pre-HSCs by measuring donor chimerism in the recipient animals. Unlabeled adult B6 BM was used as helper (CD45.2+), and could be distinguished from host NSG recipients (CD45.1+). As the injection into the facial vein of neonatal mice is technically challenging, the inclusion of helper BM (which also contains HSCs) also served as an internal control to determine injection success.

### All Pre-HSCs Are Within the CD11a- Fraction of Progenitors in e10.5 and e11.5 Embryos

Our previous study identified a population that contained all *in vitro* clonal multipotent activity in the early embryo (Inlay et al., 2014). These cells are within the CD144+ CD11a- fraction of “eKLS” cells (embryonic equivalent of the adult KLS population), which is defined as Ter119- CD43+ Kit+ Sca1+. To determine whether CD144+ CD11a- eKLS cells (“11a- eKLS”), contains pre-HSCs, we sorted and transplanted embryonic progenitors into neonatal recipients. Due to the low engraftment rate of e9.5 whole-tissue transplants (Figure 1C and Supplementary Figure 1A), we focused only on e10.5 and e11.5 tissues. We also sorted all other eKLS cells (not CD11a- CD144+ eKLS cells, or “Other eKLS”), to ensure that other potential sources of pre-HSCs were also examined. We sorted “11a- eKLS” from one color of embryo (e.g., Tomato+), and mixed it with “Other eKLS” sorted from the other color embryo (e.g., CFP+) and co-transplanted them along with helper BM into neonatal NSG recipients (Figures 2A,B and Supplementary Figure 3). We maintained the physiological ratios of the two populations, such that each recipient contained the equivalent of all eKLS cells from each embryo (Figure 2B). Thus, whichever fraction contained the most pre-HSCs would display greater donor chimerism in the recipients, regardless of how many non-pre-HSCs were contained in that fraction. On average, each recipient received 3–4 embryo equivalents (ee) of each population.

Blood analysis of recipients showed higher total CD45+ leukocyte chimerism (total blood chimerism) from the 11a- eKLS population compared to the “Other eKLS” source at both e10.5 and e11.5 timepoints and from all embryonic tissues examined (Figure 2Ci and Supplementary Figure 4). Within the short-lived granulocyte compartment, we found that only the 11a- eKLS cells gave rise to donor granulocytes in all recipients (Figure 2Cii and Supplementary Figure 4). BM analysis of recipients confirmed the presence of embryo-derived HSPCs (hematopoietic stem/progenitor cells, Ter119- CD27+ Kit+ Sca1+) only from the 11a- eKLS population with no contribution from the other eKLS source (Figure 2Ciii). We then performed secondary transplants and confirmed long-term engraftability of 11a- eKLS-derived HSCs (Figure 2Civ). We also examined the distribution of donor lineages to determine whether 11a- eKLS cells exhibited any lineage biases depending on which tissue they were sorted from Supplementary Figures 4C,D. While some recipients displayed a minor increase in T cells over B cells, there did not appear to be a consistent bias for any specific tissue source or timepoint. Notably, 11a- eKLS cells from e11.5 FL had a significantly decreased myeloid output relative to lymphoid output, due to increase T cell production. We also examined lineage output over time, but observed no trends that would indicate some tissues produce some lineages earlier or faster than others (data not shown).

While our data shows that up to e11.5, all pre-HSC activity is in the CD11a- fraction, at later timepoints, e13.5 and e14.5, we observed neonatal engraftment from both CD11a- and CD11a+ progenitors (Supplementary Figure 5A). At e14.5, we also observed adult engraftment from both CD11a- and CD11a+ fractions (Supplementary Figure 5B). This is consistent with our previous studies where we found multipotency within both CD11a- and CD11a+ fractions at e12.5 (Inlay et al., 2014), and found both CD11a- and CD11a+ fetal HSCs at e17.5 (Fathman et al., 2014). This suggests that pre-HSCs/HSCs transiently upregulate CD11a during their maturation from pre-HSCs to HSCs.

### All Pre-HSCs Are Sca1+ at e11.5 and Efficiently Identified by Anti-Sca1 Staining

**FIGURE 3:**
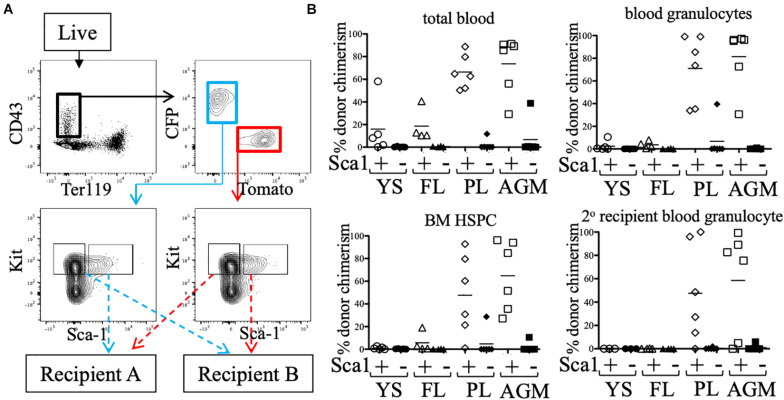
Sca1 “plus/minus” sort and neonatal transplant. **(A)** Representative FACS plot of sorted populations for transplantation (AGM shown). Live cells from e11.5 embryonic tissues were first gated on CD43+ Ter119-, then separated into CFP+ and Tomato+ fractions. Kit+ cells within these gates were sorted into Sca1+ and Sca1- fractions. Prior to transplantation into neonatal NSG recipients, cells were mixed such that Sca1+ fraction of one color (e.g., CFP) was co-transplanted with the Sca1- fraction of the other color (e.g., Tomato). **(B)** Total blood (CD45+ cells), blood granulocyte, and BM HSPC chimerism of the Sca1+ (white symbols) and Sca1- (black symbols) fractions of e11.5 embryos are shown at week 12. Week 12 bone marrow was harvested and transplanted into lethally-irradiated B6 adult recipients and blood granulocyte chimerism was analyzed 6 weeks post-secondary transplant.

Some groups have reported low/undetectable Sca1 protein expression on early hematopoietic progenitors (De Bruijn et al., 2002), and require a Sca1-GFP transgenic reporter (Ly-6A-GFP) in order to identify Sca1+ cells in the embryo. To rule out whether any pre-HSC activity was present in the Sca1- fraction, e11.5 CFP/Tomato embryos were separated into individual tissues (YS, FL, PL, and AGM) and stained (Figure 3). CD43+ Ter119- Kit+ cells were separated into Sca1+ and Sca1- fractions (from different reporters) and co-transplanted into neonatal NSG recipients (Figure 3A). 12 weeks after transplantation, we found that only the Sca1+ fraction consistently produced donor chimerism (blood granulocytes and BM HSPCs) in the recipient mice, including in secondary recipients, indicating that few, if any, pre-HSCs are contained in the Sca1- population in e11.5 tissues (Figure 3B). It should be noted that in this set of experiments, not all tissues engrafted equally, and the PL and AGM eKLS populations had higher overall chimerism than that from the YS and FL.

### CD11a- Embryonic Progenitors Are More Quiescent Compared to Their CD11a+ Counterparts

**FIGURE 4:**
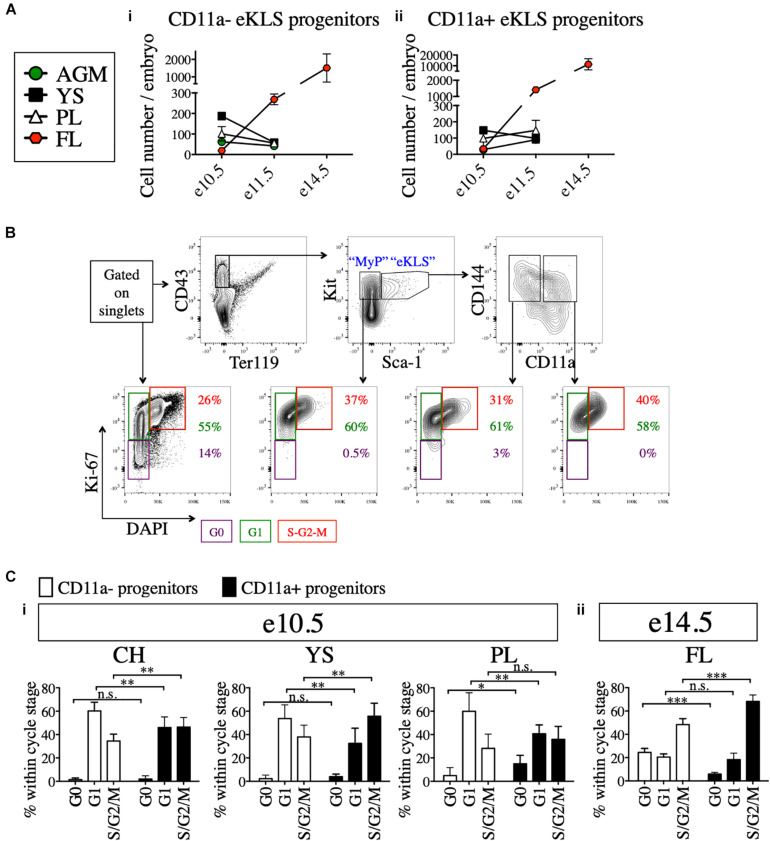
Cell cycle analysis of 11a- eKLS cells and CD11a+ progenitors. **(A)** Numbers of CD11a- and CD11a+ progenitors in embryos over time. Estimated number of CD11a- progenitors **(i)** and CD11a+ progenitors **(ii)** per embryo is depicted in embryonic tissues at e10.5, e11.5, and e14.5. “eKLS progenitors” are defined as Ter119- CD43+ Sca1+ Kit+ CD144+ at e10.5 and e11.5, and as Ter119- CD43+ Sca1+ Kit+ EPCR+ at e14.5. e10.5, *n* = 4 (two independent experiments); e11.5, *n* = 5 (three independent experiments; e14.5, *n* = 10 (two independent experiments). **(B)** Representative analysis of the cell cycle status in embryonic population. Bottom plots show DAPI (x-axis) and Ki-67 (y-axis) within total single cells, myeloid progenitors (MyP), 11a- eKLS, and 11a+ eKLS from left to right. The color of percentage values inside the gates correlate with the color of each gate/cell cycle phase. e10.5 CH is shown as a representation. **(C)** Cell cycle analysis of CD11a- and CD11a+ progenitors at e10.5 and e14.5. Cell cycle status of each population is depicted for e10.5 tissues **(i)** and e14.5 FL **(ii)**. “Progenitors” are defined as Ter119- CD43+ Sca1+ Kit+ CD144+ at e10.5, and as Ter119- CD43+ Sca1+ Kit+ CD150+ at e14.5. ^∗^*p* ≤ 0.05, ^∗∗^*p* ≤ 0.01, and ^∗∗∗^*p* ≤ 0.001 (Student’s unpaired *t* test) e10.5, *n* = 8 (two independent experiments); e14.5 = 5 (two independent experiments).

Next, we examined the absolute numbers of CD11a- and CD11a+ eKLS progenitors at different embryonic stages and in different tissues. At e10.5, both populations were most abundant in the YS, followed by the PL and AGM, with the fewest found in the FL. However, by e11.5, both populations were most abundant in the FL, and reduced in the other tissues (Figures 4Ai,ii). These data support the previously described migration of hematopoietic progenitors from the AGM, YS, and PL to the FL over time and confirm the FL as the primary site of hematopoiesis in mid-gestation (Kumaravelu et al., 2002). Furthermore, a much higher frequency of CD11a+ eKLS cells in e14.5 FL suggested the higher expansion rate of CD11a+ progenitors and/or less frequent division of the CD11a- progenitors.

We next examined the cell cycle status of early (e10.5) and late (e14.5) embryonic progenitor (eKLS) fractions (Figure 4B), using staining for the proliferation marker Ki-67 and the nuclear dye DAPI, which collectively can distinguish cells in G0, G1, and S/G2/M phases (Kim and Sederstrom, 2015). In e10.5 tissues, we observed a shift in the fraction of cells in G0/G1 phase vs. S/G2/M phase between CD11a- and CD11a+ eKLS cells respectively, suggesting a higher rate of division among the CD11a+ fraction (Figure 4Ci). This difference was more pronounced in e14.5 FL tissues, as many CD11a- eKLS cells were in G0 phase while the CD11a+ fraction had increased S/G2/M phase cells (Figure 4Cii). These observations support the notion that the CD11a- and CD11a+ embryonic progenitors begin to resemble quiescent adult HSCs and downstream transit-amplifying cells, respectively.

### The Neonatal Liver Harbors Transplanted Embryonic Progenitors Shortly After Transplant

**FIGURE 5:**
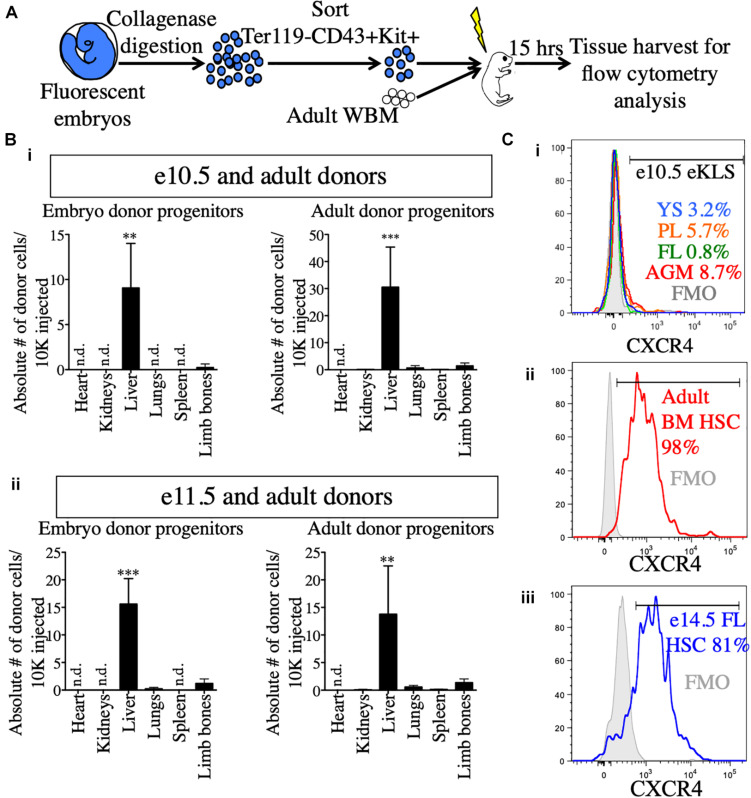
Neonatal liver harbors transplanted embryonic and adult donors in the short-term post-transplant. **(A)** Schematic representation of short-term homing assay. Ter119- CD43+ Kit+ progenitors were sorted from whole embryos, mixed with 100,000 adult WBM and transplanted into irradiated P1-P4 neonatal recipients. 15 h post-transplant, organs were harvested for FACS analysis. **(B)** Detection of donor progenitors shortly after transplant. Absolute number of Ter119- CD43+ Kit+ progenitors originating from e10.5 **(i)** and e11.5 **(ii)** embryos and the accompanying adult source normalized to 10,000 injected cells are shown in the heart, kidneys, liver, spleen, and limb bones of the neonatal recipients. Tissues were harvested and processed in their entirety. “Limb bones” includes femurs and tibias from the hind limbs and humeri from the forelimbs. ^∗∗^*p* ≤ 0.01 and ^∗∗∗^*p* ≤ 0.001 (Student’s unpaired *t* test). e10.5, *n* = 3 (two independent experiments); e11.5, *n* = 4. **(C)** Surface expression of CXCR4 in e10.5 pre-HSCs **(i)**, adult BM HSCs **(ii),** and e14.5 FL HSCs **(iii)**. Percentages of CXCR4+ cells are shown in histograms. FMOs were used to set the positive gate. Adult BM and e14.5 FL HSCs were stained and analyzed in the same experiment. e10.5 pre-HSCs were analyzed in a separate experiment alongside appropriate positive staining controls (adult BM HSCs, data not shown). FMO, fluorescence minus one; n.d., not detected.

Why do pre-HSCs engraft in neonates, but not adult recipients? Previous studies have suggested a role for the neonatal liver in providing a niche for the maturation of pre-HSCs prior to BM seeding (Arora et al., 2014). To determine whether embryonic progenitors seeded the FL directly, we transplanted e10.5 and e11.5 sorted hematopoietic progenitors (Ter119- CD43+ Kit+) along with adult BM into NSG neonates and analyzed recipient tissues 15 h post-transplant for the presence of transplanted cells (Figure 5A and Supplementary Figure 6). Amongst the different tissues examined, the liver of the recipients contained by far the highest number of transplanted progenitors originating from both e10.5 and e11.5 embryonic sources as well as the adult donor source (Figure 5B). Similar results were found when we investigated the presence of embryo-derived donor leukocytes (Ter119- CD43+) and donor eKLS (Supplementary Figure 6). These results suggest that pre-HSCs and other Kit+ progenitors may be unable to migrate directly to the BM upon transplantation, but instead first seed the FL.

To that end, we also investigated the expression of CXCR4, a receptor on HSCs known to be involved in homing to the BM on adult HSCs and embryonic pre-HSCs (Wright et al., 2002). CXCR4 was expressed on a very low percentage of e10.5 11a- eKLS cells regardless of tissue, whereas it was expressed on nearly all adult HSCs (Figures 5Ci,ii). At e14.5, the FL contains adult-engraftable phenotypic HSCs (defined as CD150+ CD48- KLS). These HSCs also expressed CXCR4 on their surface to a similar degree as adult HSCs (Figure 5Ciii). This suggests that pre-HSCs are unable to directly home to the BM in part because they lack CXCR4 expression. Together, these results suggest that the neonatal liver may serve as the initial site of engraftment of transplanted embryonic cells until their eventual maturation and seeding of the BM.

## Discussion

The precise surface marker identity of pre-HSCs in the embryo has remained elusive, hampering efforts to understand how HSCs arise during embryonic development. Our previous results identified a candidate pre-HSC population using an *in vitro* multipotency assay (Inlay et al., 2014). Here, using an *in vivo* neonatal transplantation assay, we demonstrate that this population, which we call “11a- eKLS” (Ter119- CD43+ Kit+ Sca1+ CD144+ CD11a-), contains all *in vivo* pre-HSC activity at gestational days e10.5 and e11.5 in the mouse embryo. As we compared 11a- eKLS cells to all other KLS cells in the embryo (Figure 2), as well as all Kit+ cells (Figure 3), we are confident 11a- eKLS cells are the *only* source of pre-HSCs at these timepoints. Additional markers may subdivide this population further, but 11a- eKLS cells also express CD41, CD105, and Tie2, and lack expression of Fcγr, CD11b, and Flk2 (Inlay et al., 2014). Importantly, the lack of CD11a expression on these cells was critical for distinguishing them from downstream progenitors, and could not be replaced with other pre-HSC markers such as VE-Cadherin (CD144), CD41, or CD45. While CD27 has been found to be expressed on Type II pre-HSCs in the e11.5 AGM (Li et al., 2017), we have not found CD27 to be expressed on CD11a- eKLS cells (previous unpublished observations). CD27 expression is rare in the embryo during these stages, and may upregulate later in development to mark more mature pre-HSCs. Additionally, our analysis of the cell cycle status of these populations (Figure 4) supports the notion that CD11a- eKLS cells contain pre-HSCs that slowly transition to quiescent HSCs, while the CD11a+ fraction represents downstream transit-amplifying cells.

Many groups studying embryonic origins of HSCs have found success in identifying embryonic cells with HSC potential by first culturing them *ex vivo* in cytokine combinations that drive hematopoietic maturation (Rybtsov et al., 2014). We chose an *in vivo* approach to identify cells with neonatal engraftability as an alternative, independent method of identifying HSC precursors. While primitive hematopoiesis is known to begin at e7.0–e7.5, and definitive hematopoiesis at 8.5, we did not identify neonatal engraftable cells until e9.5. Furthermore, we only identified this from unsorted caudal half tissue, and could not consistently achieve neonatal engraftment from sorted cells at e9.5. While we could identify 11a- eKLS cells at e9.5 (Inlay et al., 2014), we cannot claim this population is neonatal engraftable until e10.5.

We found 11a- eKLS cells (and its pre-HSC activity) in all tissues we examined, and thus this study is agnostic as to which embryonic tissue(s) produce pre-HSCs. Due to the challenges and highly variable engraftment rates in the neonatal transplantation system, we did not attempt to compare engraftment levels of sorted pre-HSCs between each tissue to determine which tissue contained more pre-HSCs. Unfortunately, we were unable to consistently sort enough 11a- eKLS cells from the vitelline vessels to transplant them, though they are likely an important source of pre-HSCs (de Bruijn, 2000). Vitelline vessels were carefully excluded from the YS dissections to avoid any vitelline-derived pre-HSCs from being included in the YS analyses. It should also be noted that at e9.5, only the unsorted cells from the caudal half (CH) engrafted long-term (Figure 1C). In the sorted experiments, we more often observed higher chimerism from AGM than from the YS (Figure 2C). Lastly, in the Sca1 plus/minus transplants (Figure 3B), we observed robust engraftment only from the Sca1+ (CD43+ Ter119- Kit+) cells sorted from the AGM and placenta, but not the YS or FL. Thus, while we observed engraftment from 11a- eKLS cells regardless of which timepoint or tissue we sorted them from, those sorted from the AGM tended to lead to higher engraftment. It is possible that 11a- eKLS cells are distinct from one another depending on which tissue they originated in. There appeared to be some differences in lineage distribution amongst the 11a- eKLS derived cells in the recipients (Supplementary Figure 4), most notably an increase in T cells in several recipients. However, given that the recipients are immunodeficient and devoid of their own lymphocytes, the expansion of T cells in these recipients after transplantation may be due to variability in their reconstitution kinetics and not due to intrinsic lineage biases *per se*. We had hypothesized that extra-embryonic sources of pre-HSCs (YS, PL) may contain a myeloid bias compared to embryonic sources (AGM, FL), but our results do not support that. Given the variability in lineage output in the recipients, it is possible that lineage biases exist between pre-HSCs derived from different tissues, but our assay was not sensitive enough to detect it. Alternatively, the pre-HSCs emerging from different tissues may have initial differences that disappear as these cells migrate to secondary sites such as the fetal liver and BM and mature there.

Whether fully-functional HSCs emerge *de novo* from hemogenic endothelium (HE) is unclear. Our results support the notion that a precursor emerges first from HE and then later matures into HSCs. This may require migration to a different site in order to complete development. As part of the LFA-1 complex, CD11a is involved in the extravasation of circulating immune cells. Thus, the differentiation of a pre-HSC (CD11a-) to a downstream transit amplifying progenitor (CD11a+) could be associated with a transition to a more migratory state. Interestingly, CD11a upregulation correlates with the downregulation of VE-Cadherin (CD144), a molecule necessary for forming junctions between endothelial cells. Downregulation of VE-Cadherin could allow pre-HSCs to detach from the endothelium and enter circulation while CD11a upregulation signals their extravasation to other tissues. In support of this concept, we have observed CD11a+ pre-HSC at e14.5 (Supplementary Figure 5), and CD11a+ fetal HSCs at e17.5 (Fathman et al., 2014). CD11a upregulation could be coupled to the migration of pre-HSCs into the fetal liver, and HSCs into the newly-formed BM cavity. However, it should be noted that chimerism was always lower from the CD11a+ fraction than CD11a-, suggesting CD11a+ pre-HSCs are a rare and transient population.

Why can pre-HSCs engraft in neonatal recipients, but not adult? The liver persists as an active site of hematopoiesis until shortly after birth (up to 3 weeks) (Bowie et al., 2007). As such, the neonatal liver might provide a temporary and readily accessible niche for transplanted pre-HSCs. Indeed, we found that almost all transplanted embryonic progenitors homed to the liver of neonatal recipients shortly after transplantation (Figure 5). We also observed that adult HSCs also preferentially seeded the neonatal liver over the BM, suggesting a more passive mechanism for liver seeding rather than direct homing (Figure 5B). Regardless, this suggests that the developing liver microenvironment provides pre-HSCs with maturation signals required for eventual BM homing/engraftment, such as the upregulation of the BM homing receptor CXCR4. Indeed, the upregulation of CXCR4 in the FL from e10.5 to e14.5 suggests the ability to respond to BM homing signals (CXCL12) correlates with the maturation of pre-HSCs into BM-engraftable HSCs. In this regard, many previous studies have highlighted the supportive effects of the FL niche stroma on HSC maintenance/expansion (Moore et al., 1997; Martin and Bhatia, 2005; Chou and Lodish, 2010; Khan et al., 2016).

In summary, we report a highly-enriched population “11a- eKLS” that contains *all* pre-HSC activity in the embryo. Upon transplantation into neonatal recipients, these cells appear to seed the liver first, and therein mature into BM-engraftable HSCs. As such, our data implicate this population as the immediate precursor population to HSCs. CD11a negativity was critical for identifying these cells up to e11.5, and can allow for improved isolation and characterization of developing pre-HSCs. This in turn can lay the groundwork to determine the molecular cues required for maturation into HSCs. Efforts aimed to generate HSCs from pluripotent sources, although promising and improving, have failed to display robust BM engraftment of the differentiated HSCs (Riddell et al., 2014; Lee et al., 2017; Sugimura et al., 2017). Given the similarities between pluripotent source-derived HSCs and pre-HSCs (e.g., HSC-like phenotype without BM homing/engraftment potential), identification of environmental stimuli involved in pre-HSC maturation could reveal how to generate engraftable HSCs from pluripotent stem cells.

## Data Availability Statement

The original contributions presented in the study are included in the article/Supplementary Material, further inquiries can be directed to the corresponding author/s.

## Ethics Statement

The animal study was reviewed and approved by International Animal Care and Use Committee (IACUC), and University Laboratory Animal Resources (ULAR), of the University of California, Irvine.

## Author Contributions

AK, EV, and MI designed the study and all experiments. AK and EV performed all experiments, with contributions from VS, CC, KG, PN, and YG. IW and TS contributed intellectual support, experimental strategy, and unpublished data. AK wrote the first draft of the manuscript. EV provided additional writing. MI edited the manuscript. All experiments were performed in the laboratory of MI. All authors contributed to the article and approved the submitted version.

## Conflict of Interest

The authors declare that the research was conducted in the absence of any commercial or financial relationships that could be construed as a potential conflict of interest.

## Publisher’s Note

All claims expressed in this article are solely those of the authors and do not necessarily represent those of their affiliated organizations, or those of the publisher, the editors and the reviewers. Any product that may be evaluated in this article, or claim that may be made by its manufacturer, is not guaranteed or endorsed by the publisher.
