# Supplementary material for: Absence of CD11a Expression Identifies Embryonic Hematopoietic Stem Cell Precursors via Competitive Neonatal Transplantation Assay
Source: Front Cell Dev Biol. 2021 Aug 25;9:734176. doi: 10.3389/fcell.2021.734176 (PMC8425522; doi:10.3389/fcell.2021.734176)
Supplement: Supplementary file 1 [file Data_Sheet_1.PDF]

Supplementary Material

Supplementary Figures & Legends

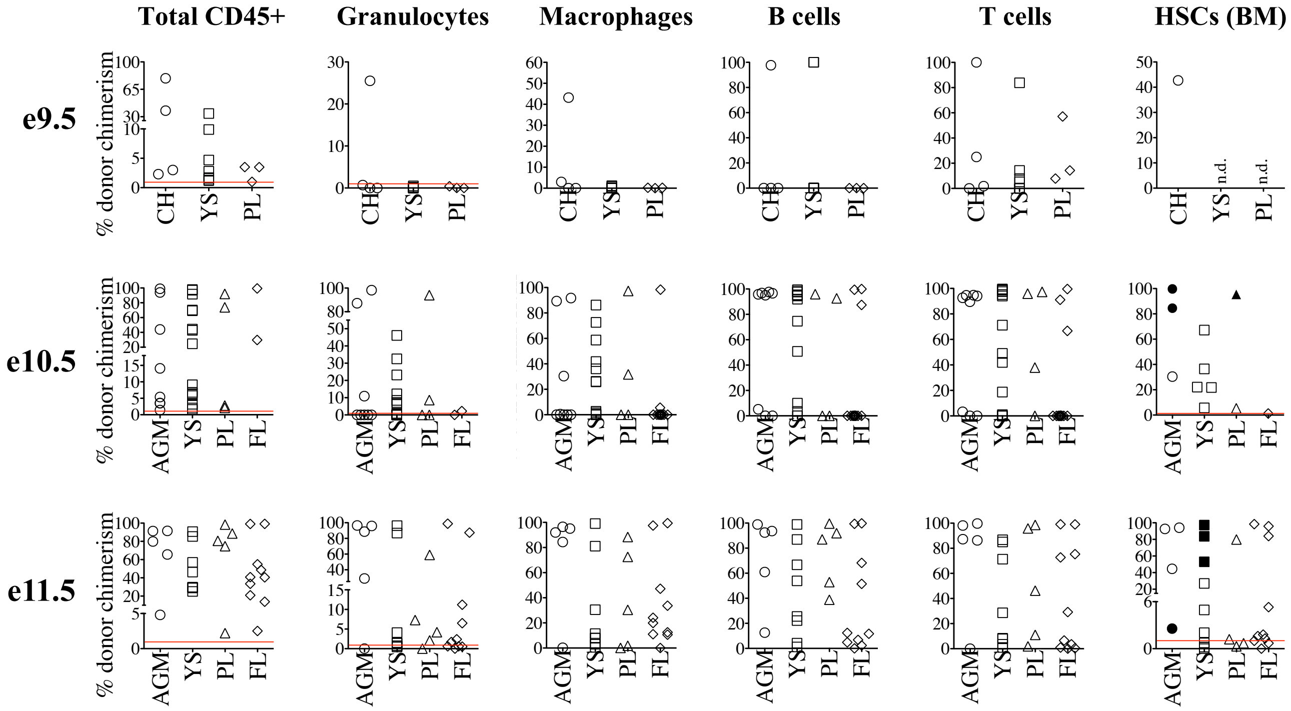

Supplementary Figure S1. Donor lineage and HSC chimerism in recipients transplanted with unsorted embryonic tissues. Donor output (% donor chimerism) in the blood of recipients transplanted with unsorted embryonic e9.5 (top row), e10.5 (middle row), and e11.5 (bottom row) tissues at the time of the last bleed (at least 12 weeks post-transplant). Percentages are relative to total output in each lineage, which includes both helper- and host-derived cells. CH = caudal half, AGM = aorta-gonad mesonephros, YS = yolk sac, PL = placenta, FL = fetal liver. Any donor chimerism (CFP+, CD45.2+) above 1% (red line) was scored as positive for that lineage. Recipients with positive donor granulocyte chimerism were further analyzed for donor-derived HSCs in the bone marrow (rightmost column). Recipients selected for secondary transplantation are indicated by closed symbols (black). See Figure 1C for a table summarizing these results. These data indicate efficient engraftment and multilineage output of e10.5 and e11.5 embryonic cells whereas e9.5 counterparts exhibited very low engraftment efficiency. *n.d. = not detected.*

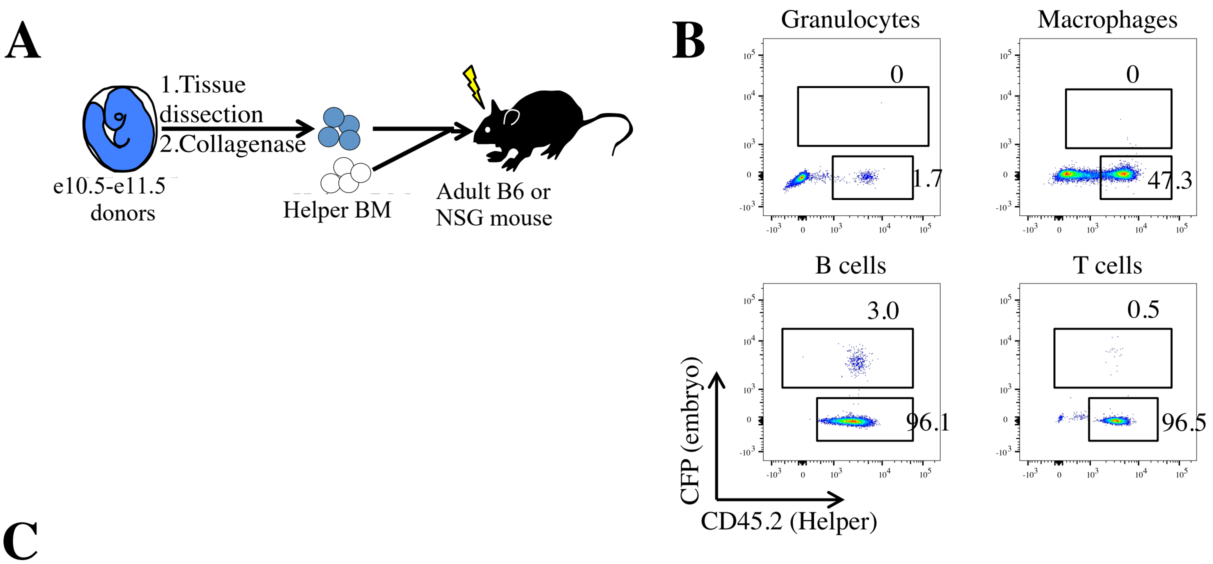

C

dpc	ee/ recipient	Adult recipient genotype	LT-chimerism	Total blood chimerism	Macrophage chimerism	B cell chimerism	T cell chimerism	Total successful transplants
e10.5	3-4	NSG	0	2	n.d.	n.d.	n.d.	3
		B6	0	0	0	0	0	5
e11.5	1-2	NSG	0	3	1	2	3	4
		B6	0	1	0	1	0	6

Supplementary Figure S2. Lack of engraftment of e10.5 and e11.5 tissues into adult recipients. **A**) Experimental strategy. Whole embryos (CFP+) were dissociated in collagenase and transplanted along with helper bone marrow (50,000 helper cells for e10.5, 100,000 helper cells for e11.5) into lethally-irradiated adult B6 and NSG recipients. **B**) Representative blood analysis at 12 weeks post-transplant. Embryo donor-derived cells are CFP+ and CD45.2+, helper-derived cells are CFP- and CD45.2+, and host cells are CFP- and CD45.2-. **C**) Table summarizing results. *n.d. = not detected.* LT-chimerism is defined as recipients with donor-derived HSCs in the BM at 12 weeks. Successful transplants are those with donor chimerism in any lineage (either embryo-derived or helper-derived), and is used to indicate mice that were "successfully" injected. While limited donor chimerism of specific mature cell types was detected, embryonic tissue-derived HSCs were not detected in the BM of any recipient, suggesting that BM-engraftable HSCs had not yet matured at these embryonic stages. This supports the notion that the neonatal engraftment observed from e10.5 and e11.5 tissues came from pre-HSCs, and not HSCs.

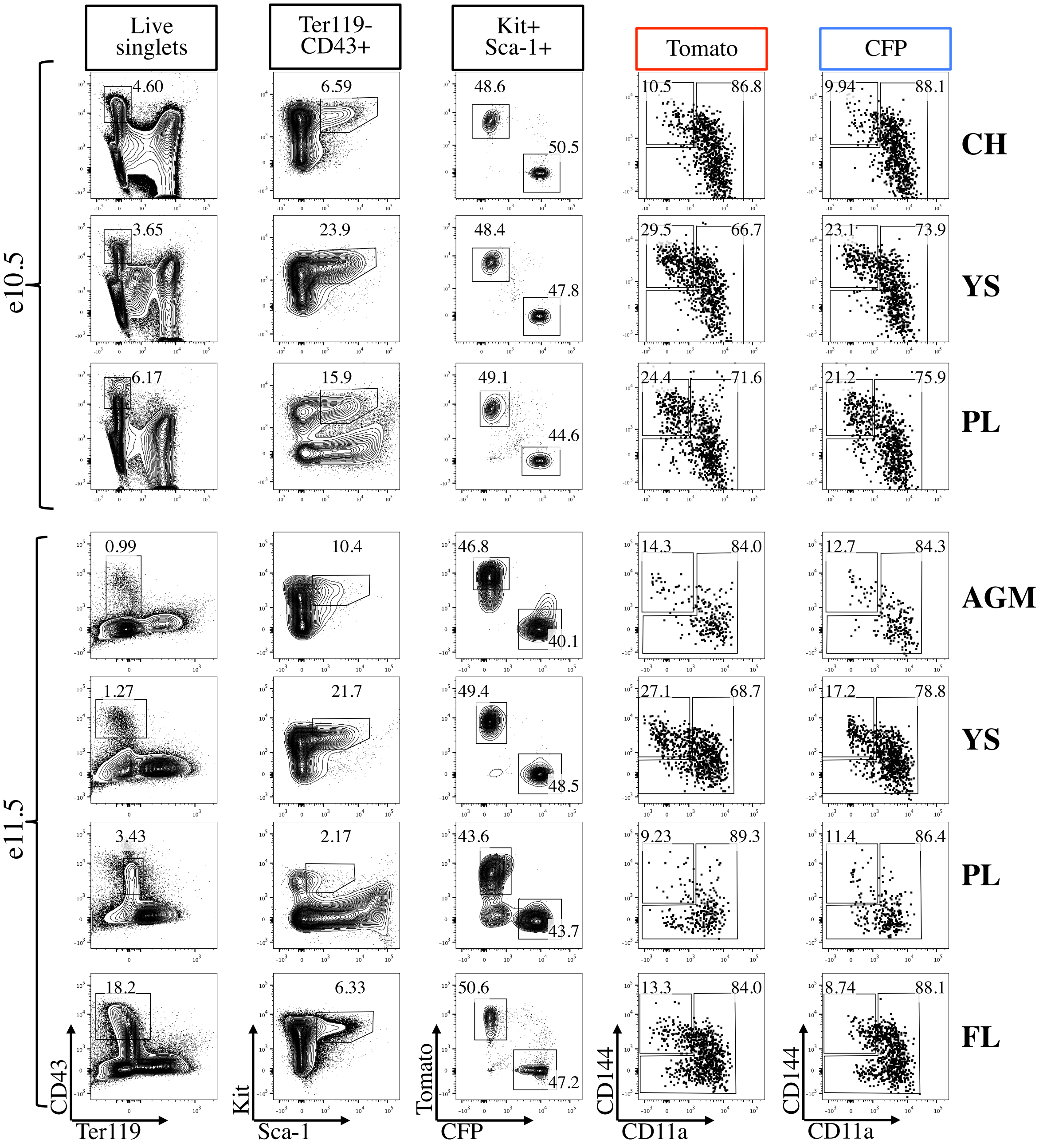

Supplementary Figure S3. Representative gating for competitive sort. A) Representative sorting strategy for the competitive transplantation of 11a- eKLS and Other eKLS populations. Each column represents the boxed population at the top of the column, and gated populations are shown to the right side of parent gates. Tissues of origin are indicated on the right side. Live Ter119- CD43+ Kit+ Sca-1+ cells are gated on based on Tomato or CFP fluorescence. Within each color of the progenitor population, CD11a- CD144+ ("11a- eKLS") and everything else ("Other eKLS") are sorted. Opposing populations of different color (i.e. CFP+ 11a- eKLS and Tomato+ Other eKLS) are mixed post-sort and transplanted into the same recipient along with helper adult BM.

A

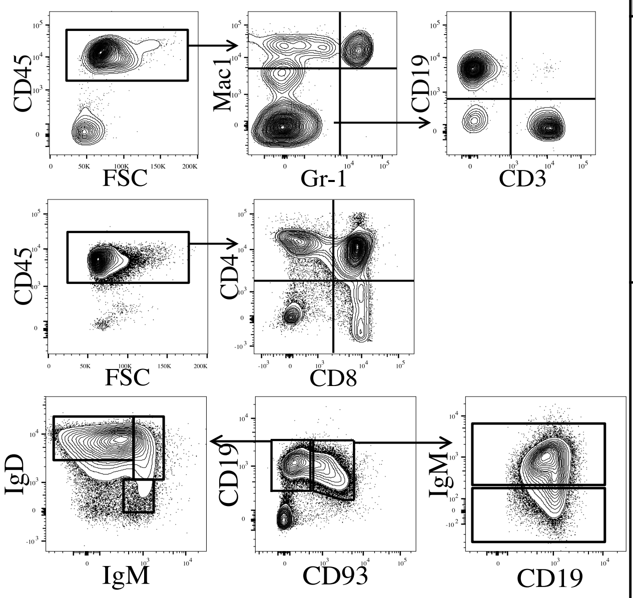

B

dpc	tissue	donor pop.	Granu-locyte chim.	Macro-phage chim.	B cell chim.	T cell chim.	total blood chim.	Success-ful inj. #
e10.5	CH	11a- eKLS	5	6	8	6	8	12
		Other eKLS	0	0	0	0	0	
	YS	11a- eKLS	3	4	6	6	6	12
		Other eKLS	0	0	1	2	2	
	PL	11a- eKLS	3	4	4	5	3	7
		Other eKLS	0	0	0	0	0	
e11.5	AGM	11a- eKLS	4	4	4	4	4	6
		Other eKLS	0	0	0	1	2	
	YS	11a- eKLS	5	5	8	7	6	7
		Other eKLS	0	0	1	1	3	
	PL	11a- eKLS	6	7	7	7	7	8
		Other eKLS	0	1	2	2	3	
	FL	11a- eKLS	5	6	7	7	7	8
		Other eKLS	0	1	4	4	3	

C D

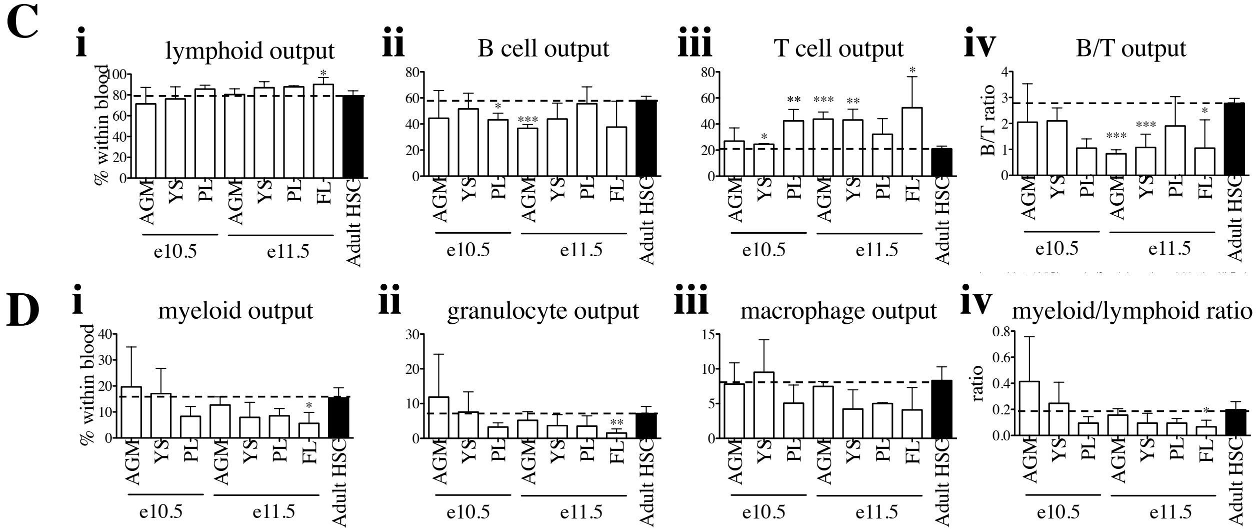

Supplementary Figure S4. Lineage output dynamics of 11a- eKLS embryonic donors in neonatal recipients. **A)** Representative FACS plots of donor lineage output in neonatal recipients at the final timepoint in blood (top row), thymus (middle row), and spleen (bottom row). All gates are pregated on donor embryo-derived cells. For the blood, macrophages (CD11b+ Gr1-), granulocytes (CD11b+, Gr1+), B cells (CD19+) and T cells (CD3+) represent the gates used for the blood analyses throughout the study. Analysis of T cell populations in the thymus, and B cell subpopulations in the spleen demonstrate that donor hematopoiesis appears normal in these tissues. **B**) Table summarizing results in Figure 2C. The number of recipients with donor lineage chimerism (chim.) and total donor CD45+ chimerism (total blood chim) are indicated. The number of mice that displayed either donor embryo or helper chimerism greater than 1% are considered successfully injected is indicated in the rightmost column (successful inj. #). While many conditions (population/timepoint/tissue) supported lymphocyte reconstitution, myeloid reconstitution was only observed with 11a- eKLS donor cells, and

never with "other eKLS" cells. This demonstrates that pre-HSCs are only found in the 11a- eKLS population. **C**) Lymphoid lineage analysis at 12 weeks post-transplant of CD11a- eKLS cells from e10.5 and e11.5 tissues. **i**) donor lymphoid output (B cells and T cells) as a percentage of total donor CD45+ cells, from different 11a- eKLS sources. Only the e11.5 FL showed a statistically significant difference, albeit mild, in lymphoid output compared to recipients transplanted with adult HSCs. This suggests there does not appear to be a bias towards or against lymphocyte production or homeostasis with embryonic donors. Output specifically in B cells (**ii**) and T cells (**iii**) is shown as well as the ratio of B:T cells (**iv**). The dashed horizontal bar represents the output from adult recipients transplanted with adult HSCs (black bar), which was used for comparison to represent normal hematopoietic constitution. All statistical analyses are relative to those recipients. There appears to be a statistically significant increase in T cell output relative to B cells with several of the embryonic tissues, but is inconsistent and could be due to the variability in lymphopoiesis that occurs in NSG recipients, which lack lymphocytes and therefore undergo a rapid expansion of lymphocytes. **D**) Donor myeloid output in recipient mice. Total myeloid output (**i**), as well as specific granulocyte (**ii**) and macrophage (**iii**) are shown. The ratio of myeloid to lymphoid cells is shown. Only the e11.5 FL displayed a statistically significant decrease in myeloid cells relative to lymphoid compared to the adult HSC transplanted controls. This could be due to a burst of lymphopoiesis in NSG recipients, which lack lymphocytes of their own. This data shows that there does not appear to be a lineage bias of 11a- eKLS cells depending on which tissue they are obtained from.

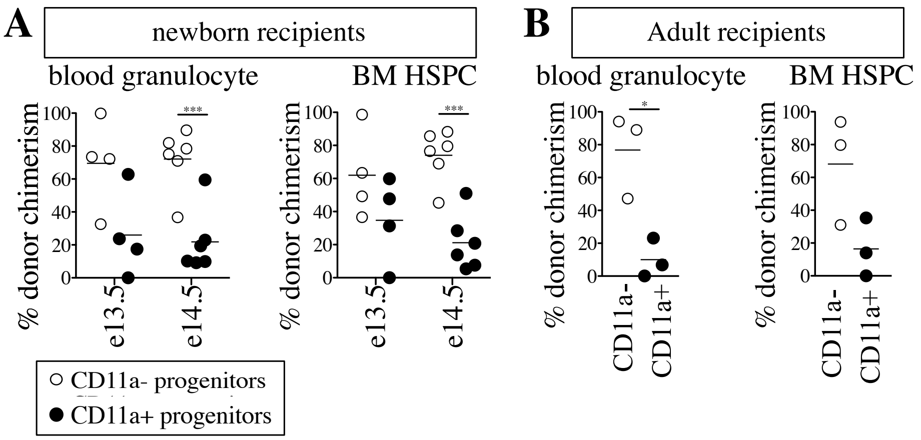

Supplementary Figure S5. At later timepoints (e13.5 and e14.5), both CD11a- and CD11a+ cells are capable of neonatal and adult engraftment. Long-term engraftment of CD11a- and CD11a+ progenitors from e13.5-e14.5 FL in neonatal recipients (**A**) and from e14.5 FL in adult recipients (**B**). "Progenitors" are defined as Ter119- CD43+ Sca1+ Kit+ EPCR+. Blood granulocyte chimerism and BM HSPC chimerism are shown for each set of recipients. These results demonstrate that at later embryonic timepoints, neonatal engraftable pre-HSCs and bone marrow engraftable HSCs begin to upregulate CD11a. **p* ≤ *0.05, ***p* ≤ *0.001 (Student's unpaired t test). Each condition (e13.5 or e14.5 transplanted into neonatal or adult recipient) performed in 2 independent experiments.*

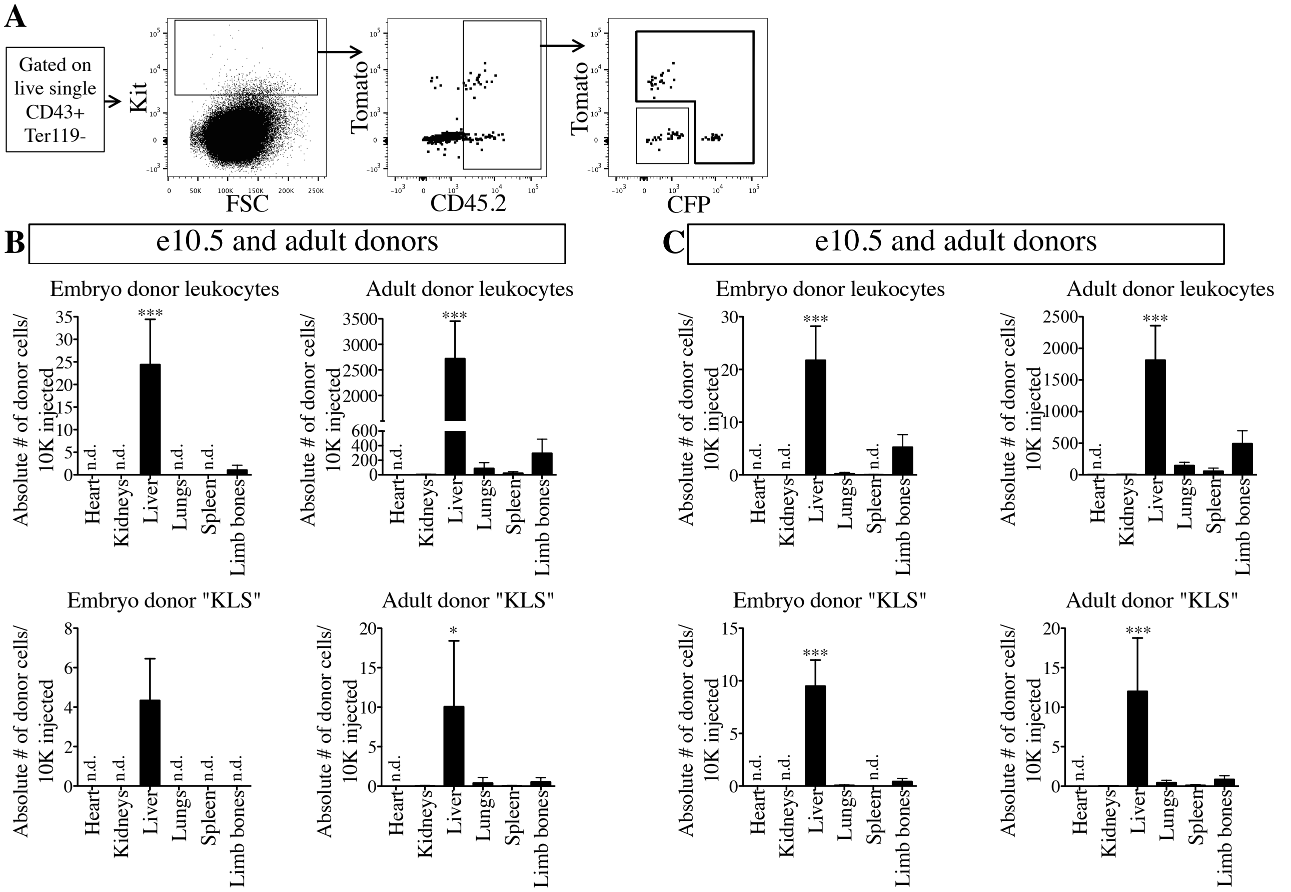

Supplementary Figure S6. Detection of embryo donor- and adult donor-derived populations in neonatal recipients shortly after transplantation. A) Representative analysis of recipient tissues in short-term homing assays. Embryo donors are identified as CFP+ or Tomato+ cells expressing CD45.2+ within the population of interest (in this case, Ter119- CD43+ Kit+). Adult WBM is distinguished by the expression of CD45.2 (recipient NSGs exclusively express CD45.1) along with lack of fluorescence (fluorescent proteins are specific to embryos). Although rare, donor cells were readily identifiable in distinct populations. **B-C)** Detection of e10.5 **(B)** and e11.5 **(C)** donor cells shortly after transplant. "Donor leukocytes" is defined as Ter119- CD43+ and "donor KLS" is defined by Ter119- CD43+ Kit+ Sca-1+. ***p ≤ 0.01, ***p ≤ 0.001 (Student's unpaired t test). e10.5, n=3 (2 independent experiments); e11.5, n=4. n.d., not detected.*

Supplementary Tables

Table S1. Antibodies Table				
**Antigen**	**Clone**	**Conjugate**	**Source**	**Catalogue #**
TER119	TER119	PE/Cy5	Biolegend	116210
	TER119	BV421	Biolegend	116233
SCA1 (Ly-6A/E)	E13-161.7	PE/Cy7	eBioscience	122514
	E13-161.7	PE	Biolegend	122507
KIT (CD117)	ACK2	APC	Biolegend	135107
	2B8	APC-eFluor 780	eBioscience	47-1171-82
	2B8	BV421	Biolegend	105828
CD27	LG.7F9	eFluor 780	eBioscience	47-0271-82
	LG.7F9	APC	eBioscience	17-0271-82
CD11A	M17/4	PE/Cy7	eBioscience	25-0111-30
	M17/4	Biotin	Biolegend	101103
	M17/4	APC	Biolegend	101119
	M17/4	PE	Biolegend	101107
	M17/4	FITC	Biolegend	101106
	M17/4	Alexa Fluor 488	Biolegend	101111
EPCR (CD201)	eBio1560	PerCP-eFluor 710	eBioscience	46-2012-82
	eBio1560	APC	eBioscience	17-2012-82
GR1 (Ly-6G/Ly-6C)	RB6-8C5	Alexa Fluor 700	eBioscience	108422
MAC1 (CD11b)	M1/70	APC	Biolegend	101212
	M1/70	FITC	Biolegend	101205
CD19	6D5	APC	Biolegend	115512
	eBio1D3	PerCP-Cy5.5	eBioscience	45-0193-82
	6D5	BV421	Biolegend	115537
CD45	30-F11	APC/Cy7	Biolegend	103116
	30-F11	Alexa Fluor 700	Biolegend	103128
CD45.2	104	FITC	Biolegend	109806
CD45.1	A20	PE/Cy7	Biolegend	110729
CD3ε	17A2	PerCP-eFluor 710	eBioscience	46-0032-82
	17A2	PE/Cy7	Biolegend	100220
CD150 (SlamF1)	TC15-12F12.2	Brilliant Violet 650	Biolegend	115931
CD4	RM4-5	PE/Cy7	Biolegend	100527
CD8a	53-6.7	APC/Cy7	Biolegend	100714
CD93 (AA4.1)	AA4.1	APC	eBioscience	17-5893-81
IgD	11-26c.2a	Alexa Fluor 700	Biolegend	405729
IgM	RMM-1	APC/Cy7	Biolegend	406515
B220	RA3-6B2	BV605	Biolegend	103243
Cxcr4	2B11	PE	BD Biosciences	561734
Ki-67	16A8	PE	Biolegend	652403
CD43	S7	APC	BD Biosciences	560663
CD144	BV13	Biotin	Biolegend	138008

Secondary antibodies				
		Qdot 655-Streptavidin	Life Technologies	Q10121MP
		Qdot 605-Streptavidin	Life Technologies	Q10103MP
		eFluor710-Streptavidin	eBioscience	49-4317-80
